# Effects of different acupuncture treatment methods on mild cognitive impairment: a study protocol for a randomized controlled trial

**DOI:** 10.1186/s13063-019-3670-3

**Published:** 2019-09-04

**Authors:** Jae-Hong Kim, Myoung-Rae Cho, Gwang-Cheon Park, Jeong-Soon Lee

**Affiliations:** 1 Department of Acupuncture and Moxibustion Medicine, College of Korean Medicine, DongShin University, Naju City, 58245 Republic of Korea; 2 Clinical Research Center, DongShin University Gwangju Korean Medicine Hospital, 141, Wolsan-ro, Nam-gu, Gwangju City, 61619 Republic of Korea; 3 Department of Nursing, Christian College of Nursing, Gwangju City, 61662 Republic of Korea

**Keywords:** Mild cognitive impairment, Acupuncture, Randomized controlled trial, Study protocol

## Abstract

**Background:**

Mild cognitive impairment (MCI) is an intermediate state between normal aging and Alzheimer’s disease, which is the world’s most common form of dementia. It is important to identify early and easily available interventions to delay the progression of MCI to Alzheimer’s disease. Acupuncture has been reported to improve the clinical outcomes of MCI treatment. Acupuncture is a complex intervention, involving both specific and non-specific factors associated with therapeutic benefits. Therefore, we intend to obtain basic data for developing an optimal acupuncture treatment for MCI by comparing the effects of different acupuncture treatment methods on cognitive function in MCI patients.

**Methods:**

This study will be a prospective, outcome-assessor-blinded, parallel-arm, single-center (DongShin University Gwangju Korean Medicine Hospital, Republic of Korea), randomized controlled clinical trial. Thirty-two participants with MCI will be randomized in equal numbers to four groups (basic acupuncture (BA), acupoint specificity (AS), needle duration (ND), or electroacupuncture (EA)) and receive acupuncture treatment once per day, 3 days/week for 8 weeks. The BA and ND groups will receive acupuncture treatment for 30 and 20 min, respectively, at Baihui (GV20), Sishencong (EX-HN1), Fengchi (GB20), and Shenting (GV24). The EA group will receive electroacupuncture treatment at the same acupoints for 30 min. The AS group will receive acupuncture treatment at GV20, EX-HN1, GB20, GV24, and Taixi (KI3) for 30 min. The outcome measured will be scores on the Korean version of the Alzheimer’s Disease Assessment Scale—cognitive subscale, the Korean version of the Montreal Cognitive Assessment, the Center for Epidemiological Studies Depression scale, the Korean Activities of Daily Living scale, the Korean Instrumental Activities of Daily Living scale, and the European Quality of Life Five Dimension Five Level scale. All scores will be recorded before intervention, 8 weeks after the first intervention, and 12 weeks after completing the intervention.

**Discussion:**

Four acupuncture protocols will be assessed and compared as potential MCI treatments. This study is expected to provide data to be used in developing an optimal acupuncture method for MCI treatment.

**Trial registration:**

Clinical Research Information Service, KCT0003430. Registered on 16 January 2019.

http://cris.nih.go.kr).

**Electronic supplementary material:**

The online version of this article (10.1186/s13063-019-3670-3) contains supplementary material, which is available to authorized users.

## Background

Mild cognitive impairment (MCI) is defined as a subjective and objective decline in cognition and function greater than expected for an individual’s age and education level, which neither meets the criteria for diagnosis as dementia nor is severe enough to interfere with activities of daily living (ADL) [1, 2]. MCI represents a significant risk factor for the development of dementia and is the primary target for early detection and management of dementia [3, 4].

Currently, there is no approved pharmacological treatment for MCI [2]. Systematic reviews and meta-analyses evaluating the efficacy of cholinesterase inhibitors (such as donepezil, rivastigmine, and galantamine) for MCI treatment have concluded that there is no convincing evidence that cholinesterase inhibitors have an effect on cognitive test scores or the progression of MCI to Alzheimer’s disease [5, 6]. There is some evidence suggesting that nonpharmacological interventions such as cognitive training [7], physical exercise [8], and acupuncture [9, 10] might be beneficial for patients with MCI. However, there is currently no established treatment method for MCI [4].

Acupuncture is a core component of traditional Chinese medicine; recent systematic reviews of the limited evidence in the literature suggest that acupuncture might improve cognitive function and ADL in patients with cognitive impairment and dementia [11–13]. However, each clinical trial to date has used a different acupuncture treatment method. Apart from needle insertion, factors such as needling sensation, psychological factors, acupoint specificity, acupuncture manipulation, and needle duration also influence the therapeutic effects of acupuncture [14].

Although acupuncture is used for MCI treatment in Korean medicine, no studies have been conducted to develop an optimal acupuncture treatment method for this condition. Therefore, through this proposed randomized controlled trial, we intend to obtain basic data for developing an optimal acupuncture method for MCI treatment.

## Methods/design

### Aims

We intend to obtain basic data for developing an optimal acupuncture treatment for MCI by comparing the effects of different acupuncture treatment methods (variable acupoint specificity, needle duration, and electrical stimulation) on cognitive function in MCI patients. Our study will indicate whether it is feasible to randomize participants to different acupuncture treatments for MCI and suggest whether acupuncture is an acceptable treatment for the included participants. The results of this study will be the basis of a future clinical trial to investigate the effects of optimal acupuncture treatment for MCI.

### Hypothesis

The null hypothesis is that randomization of patients into four equal groups each receiving a different acupuncture protocol is not feasible and/or acupuncture is not safe for MCI patients and different acupuncture treatment methods will provide similar improvements in cognitive function in MCI patients.

### Study design and setting

This study is a prospective, outcome-assessor-blinded, parallel-arm, single-center (DongShin University Gwangju Korean Medicine Hospital, Republic of Korea), randomized controlled clinical trial with a 1:1:1:1 allocation ratio. A total of 32 participants, who meet the inclusion and exclusion criteria, will be randomly allocated to a basic acupuncture (BA), an acupoint specificity (AS), a needle duration (ND), or an electroacupuncture (EA) group (*n* = 8 each). The patients will receive acupuncture treatment once per day, 3 days/week for 8 weeks.

The outcome measure will be improvement in cognitive function evaluated using the Korean version of the Alzheimer’s Disease Assessment Scale—cognitive subscale (ADAS-K-cog) and the Korean version of the Montreal Cognitive Assessment (MoCA-K), and improvement in scores on the Center for Epidemiological Studies Depression (CES-D), Korean Activities of Daily Living (K-ADL), Korean Instrumental Activities of Daily Living (K-IADL), and European Quality of Life Five Dimension Five Level (EQ-5D-5L) scales. All scores will be recorded at baseline (before intervention), 8 weeks after the first intervention (i.e., at the end of the intervention period), and 12 weeks after completion of the intervention.

The design of this study, including outcomes measured and evaluation schedules, is based on the designs of several previous studies that have evaluated acupuncture treatment for cognitive impairment [11, 12, 15]. This manuscript has been written in accordance with the Standard Protocol Items: Recommendations for Interventional Trials (SPIRIT) and Consolidated Standards Of Reporting Trials (CONSORT) 2010 guidelines [16, 17] (see Additional file 1).

**Fig. 1:**
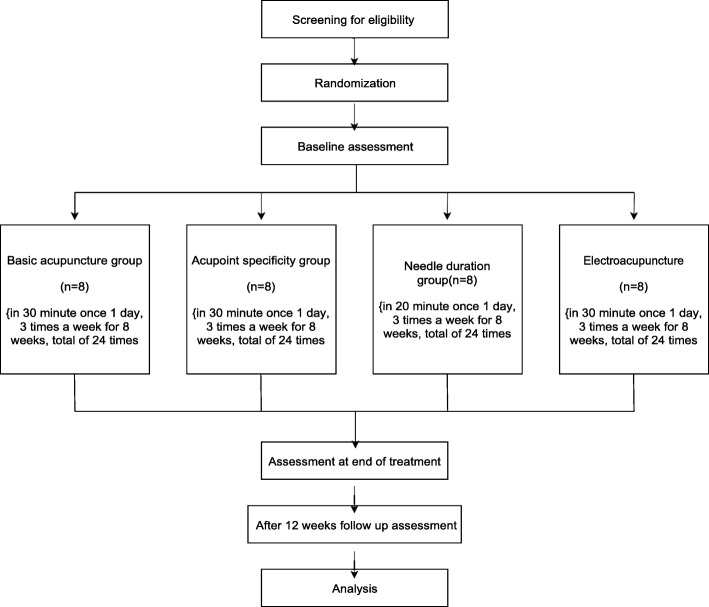
Flow chart of the trial

**Fig. 2:**
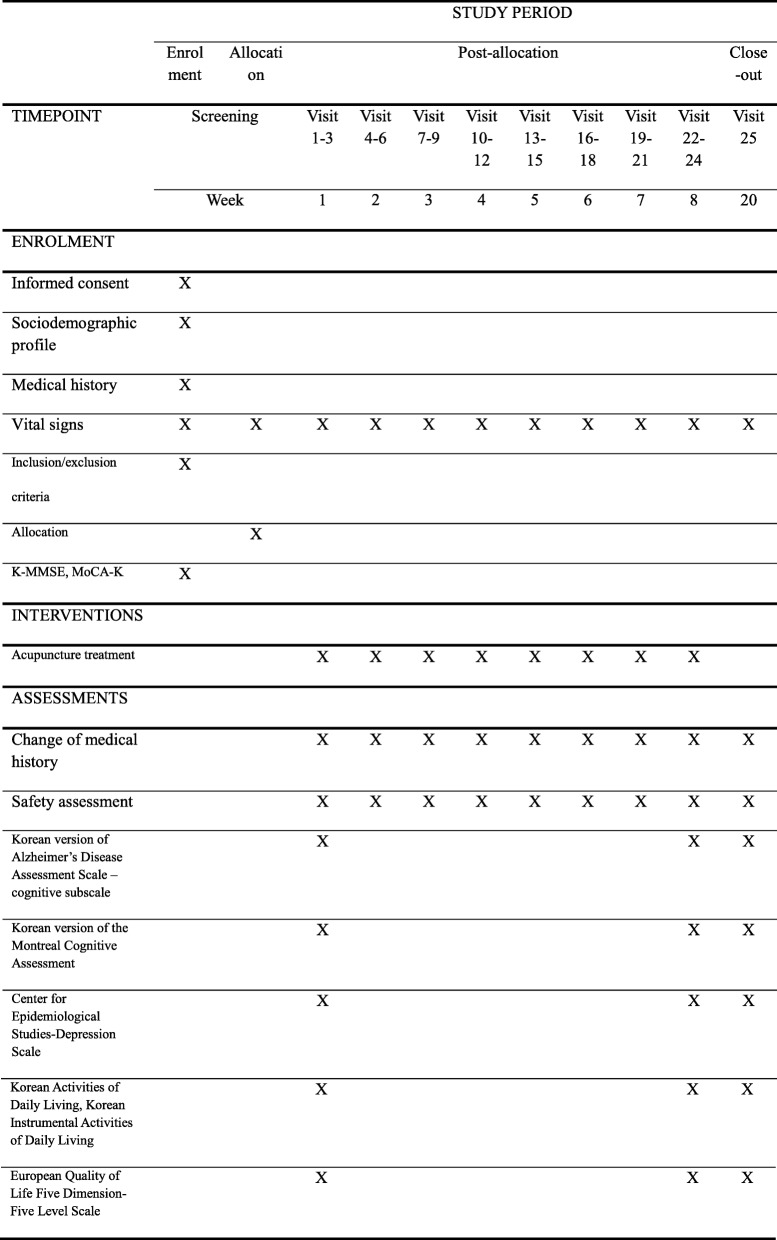
Standard Protocol Items: Recommendations for interventional Trials Statement (SPIRIT) figure showing the enrolment, interventions, and data collection

This study protocol complies with the principles of the Declaration of Helsinki and Korean Good Clinical Practice guidelines. The trial has been registered at http://cris.nih.go.kr (registration number: KCT 0003430; registration date: 16 January 2019). The study design is summarized in Figs. 1 and 2.

### Participant recruitment

Participants will be recruited at the DongShin University Gwangju Korean Medicine Hospital in the Republic of Korea. The study will be advertised through local newspapers, the Internet, and posters in communities and hospitals. Interested individuals will learn how to participate in this clinical trial through telephone calls or visits to our hospital. During visits to the clinical research center of DongShin University Gwangju Korean Medicine Hospital, the clinical research coordinator (CRC) will explain the study to the participants, who will be asked to voluntarily sign an informed consent form before participation. In order for the enrolled participant to be able to complete treatment and evaluation, the CRC will adjust the treatment and evaluation schedule of the enrolled participant to facilitate participation in this study. Every time the enrolled participant visits, the CRC will explain the next visit’s schedule; and the day before the visit, the CRC will remind the enrolled participant of the schedule by telephone. The CRC will continuously monitor the medical condition of enrolled participants to ensure adherence to the intervention protocols.

### Inclusion criteria

Participants meeting all of the following criteria will be included in this trial: male or female aged 55–85 years; fulfillment of the Petersen diagnostic criteria for MCI [1] (i.e., subjective memory complaint, normal general cognitive function, objective memory impairment, not severe enough to interfere with ADL, and no dementia) with memory impairment for at least the 3 months preceding enrollment; score of 20–23 on the Korean version of the Mini-Mental State Examination (MMSE); score of 0–22 on the MoCA-K; sufficient language fluency to reliably complete all study assessments; and willingness to sign the informed consent form.

### Exclusion criteria

The exclusion criteria are as follows: dementia diagnosed in accordance with the *Diagnostic and Statistical Manual of Mental Disorders* fourth edition; history of brain lesions that might cause cognitive impairment (e.g., traumatic brain injury, stroke, intracranial-space-occupying lesions, or congenital mental retardation); presence of cancer and/or serious cardiovascular, cerebrovascular, liver, or kidney disease; history of treatment for alcohol/drug dependency or mental diseases (e.g., schizophrenia, serious anxiety, or depression) in the 6 months preceding enrollment; current treatment for MCI (e.g., medication, acupuncture, or cognitive training); difficulties in assessment due to visual or hearing impairment; being unfit for acupuncture (e.g., blood-clotting abnormalities (e.g., hemophilia), scalp infection, or presence of a pacemaker); and concurrent participation in other clinical trials.

### Dropout criteria

Participants will be dropped from the trial under the following conditions: less than 75% compliance with the protocol procedures (i.e., participating in fewer than 18 of the 24 scheduled treatment sessions); occurrence of a serious adverse event (SAE); reluctance to continue the trial; incomplete data that could influence the trial; large error in protocol or significant deviation in implementation; or if the principal investigator (PI) or institutional review board (IRB) decide to terminate their participation in the trial.

### Ethical considerations

This study has been approved by the IRB of DongShin University Gwangju Korean Medicine Hospital, Republic of Korea (DSUOH-051). The purpose and potential risks of this clinical trial will be fully explained to the participants and their families. All participants will be asked to provide written informed consent before participation.

### Randomization

After acquiring written informed consent and completing the baseline measurements, the 32 enrolled participants will be assigned serial numbers generated by SPSS software version 21 (IBM Corp., Armonk, NY, USA) and randomly allocated to one of the four study groups (*n* = 8 each). The serial number codes will be inserted into opaque envelopes, which will be sealed and stored in a double-locked cabinet. These envelopes will then be opened in the presence of the patient and a guardian.

### Implementation

The CRC will generate the allocation sequence, enroll participants, and assign participants to interventions.

### Blinding

Because of the nature of acupuncture treatment, it is not possible to adopt a double-blinded study design. Therefore, we will adopt an outcome-assessor-blinded trial procedure. During the course of this clinical trial, the assessor will not contact any participant other than at the time of assessment. Furthermore, assessor unblinding will not be permitted under any circumstance. For preventing selection, performance, and attrition biases due to unblinded participants and practitioners, this study will only involve individuals without conflicts of interest or preconceived positions. All practitioners will receive training in clinical trials before participation. A statistician, who was not involved in the design or execution of the clinical trial, will analyze the final data.

### Interventions

Participants will receive acupuncture treatment once per day, 3 days/week (excluding Saturdays and Sundays) for 8 weeks. The treatment will be administered by Korean medicine doctors with 6 years of formal university training in Korean medicine and a license to administer treatment. To ensure strict adherence to the study protocol, the doctors will receive training together and use the same techniques (Table 1).

**Table 1:** Revised Standards for Reporting Intervention in Clinical Trials of Acupuncture (STRICTA)

Item	Item criteria	Description
1. Acupuncture rationale	1a) Style of acupuncture	Korean medicine therapy
	1b) Reasoning for treatment provided—based on historical context, literature sources, and/or consensus methods, with references where appropriate	• Discussion among four doctors that practice Korean medicine (consensus) • Textbook of acupuncture and moxibustion medicine • Relevant articles [9, 17–22] Selection of treatment regions based on textbooks, related papers, and expert discussions
	1c) Extent to which treatment varied	Standardized treatment
2. Details of needling	2a) Number of needle insertions per subject per session (mean and range where relevant)	8 or 10
	2b) Names (or location if no standard name) of points used (unilateral/bilateral)	Baihui (GV20), Sishencong (EX-HN1), Fengchi (GB20), Shenting (GV24), Taixi (KI3)
	2c) Depth of insertion, based on a specified unit of measurement or on a particular tissue level	Needles will be inserted into the acupoints subgaleally along the scalp at an angle of 15–30°. GB20 will be punctured 17–30 mm in the direction toward the nose tip. GV24, the anterior EX-HN1, and GV20 will be punctured forward, and the left, right, and posterior EX-HN1 toward GV20. The depth of insertion will be 9–24 mm depending on the location of the needle. KI3 will be punctured bilaterally, vertically to a depth of 9–15 mm [9, 23]
	2d) Responses sought	No de qi or muscle twitching—only sensation due to needle insertion
	2e) Needle stimulation	None or electrical stimulation
	2f) Needle retention time	30 min per session or 20 min per session
	2 g) Needle type	Sterile, stainless, disposable acupuncture needles (size 0.25 mm × 30 mm, product no. A 84010.02; Dong Bang Acupuncture, Inc., Boryeong, Republic of Korea)
3. Treatment regimen	3a) Number of treatment sessions	24
	3b) Frequency and duration of treatment sessions	3 days/week for 8 weeks, 30 min per session or 20 min per session
4. Other treatment components	4a) Details of other interventions administered to the acupuncture group	None
	4b) Setting and context of treatment—including instructions to practitioners—as well as information and explanations given to patients	Practitioner–patient conversation about the context of the treatment, life habits, and daily life management
5. Practitioner background	5a) Description of participating acupuncturists	Korean medicine doctor with the following qualifications: 6 years of formal university training in Korean medicine and a license
6. Control or comparator interventions	6a) Rationale for the control or comparator in the context of the research question, with sources that justify the choice	[9]
	6b) Precise description of the control or comparator; details for items 1–3 with the use of sham acupuncture or any other type of acupuncture-like control	This study will investigate the optimal acupuncture treatment method for the treatment of mild cognitive impairment (MCI) through a comparison of the effects of different acupuncture treatment methods according to acupoint specificity, needle duration, and electrical stimulation in terms of an improvement in cognitive function in patients with MCI. There is no control or comparator

### BA group

BA treatment will be acupuncture administered at Baihui (GV20), Sishencong (EX-HN1), Fengchi (GB20), and Shenting (GV24) [9, 15]. Only sterile, stainless steel, disposable acupuncture needles (size 0.25 mm × 30 mm, product no. A84010.02; Dong Bang Acupuncture, Inc., Boryeong, Republic of Korea) with guide tubes will be used for treatment. With participants in the sitting position, needles will be subgaleally inserted at an angle of 15–30° along the scalp. GB20 will be punctured 17–30 mm in the direction of the tip of the nose. GV24, anterior EX-HN1, and GV20 will be punctured in the forward direction, while the left, right, and posterior EX-HN1 will be punctured in the direction of GV20. The depth of insertion will be 9–24 mm, depending on the location of the needle [23]. After insertion, the needles will be left in position for 30 min. Manual stimulation will not be used.

### AS group

The AS acupuncture treatment method will be the same as the BA treatment method except for additional acupuncture treatment at Taixi (KI3) [18–21], where acupuncture needles will be inserted bilaterally and vertically to a depth of 9–15 mm [23] for 30 min.

### ND group

The acupuncture treatment method for the ND group will be the same as that for the BA group, except that the needles will be retained for 20 min instead of 30 min [22].

### EA group

The acupuncture treatment method for the EA group will be the same as that for the BA group, except that it will involve electrical stimulation of the acupoints using an EA stimulator (CELLMAC PLUS, STN-330, product no. A16010.04; Stratek, Co., Ltd, Anyang, Republic of Korea). GV24and GV20, the left and right EX-HN1, the anterior and posterior EX-HN1, and the left and right GB20 will be subjected to EA under the following parameters: continuous waves; frequency, 3–15 Hz; and intensity, 2–4 mA [9].

During the clinical trial period, all participants will be allowed to continue their routine management regimens (including regular physical exercise), existing medications (such as those for hypertension, diabetes, or hyperlipidemia), and medications for maintaining and improving their health status. However, they will not be permitted to engage in other treatments (e.g., pharmacological treatment or cognitive rehabilitation) for ameliorating their MCI symptoms. All medical devices, including the acupuncture needles and EA stimulator, will be inspected by the investigators, who will record their findings in the management register.

### Outcome measurements

The scores for the ADAS-K-cog, MoCA-K, CES-D, K-ADL, K-IADL, and EQ-5D-5L scales will be recorded before treatment, at the end of treatment, and 12 weeks after treatment completion.

General cognitive performance will be evaluated using the ADAS-K-cog. The ADAS-cog is a quantitative instrument for evaluating comprehensive cognitive functions involving memory, language, praxis, and frontal lobe function [24]. It is also used for judging the efficacy of anti-dementia treatments [22, 25, 26]. The scale has been translated into Korean and validated [27]. This adapted scale includes tests for word recall, naming of objects and fingers, commands, constructional and ideational praxis, orientation, word recognition, spoken language and comprehension, word finding, and recall of test instructions. Higher scores indicate higher degrees of deficit [28].

The MoCA scale is a clinician-friendly, validated, brief instrument with high sensitivity and specificity for detecting MCI [29]. It has been translated into Korean and validated [30]. Subscales of this tool include tests for evaluating overall cognitive function, including visuospatial ability, executive function, attention–concentration–working memory, language, short-term memory recall, and orientation to time and place [30].

The CES-D scale is a short self-report scale designed to measure the current level of depressive symptomatology, including depressed mood, feelings of guilt and worthlessness, feelings of helplessness and hopelessness, psychomotor retardation, loss of appetite, and sleep disturbance. Patients are asked to rate each item using an ordinal 4-point Likert scale [31].

The K-ADL and K-IADL scales will be used for assessing physical function. The K-ADL scale was developed to assess basic activities of the elderly, including dressing, washing, bathing/showering, eating, getting out of bed/the bedroom, using the toilet, and controlling urination. The K-IADL scale is used for estimating more complex activities necessary for an independent daily life, including personal grooming, household chores, preparing meals, doing laundry, going out within a short distance, using transportation, shopping, managing money, making telephone calls, and taking medications [32]. Combining IADL and ADL items on the same scale provides an enhanced range and sensitivity of measurement [33].

The European Quality of Life Five Dimension (EQ-5D) scale is a generic instrument for assessing health-related quality of life, comprising five dimensions: mobility, self-care, usual activities, pain/discomfort, and anxiety/depression. Each dimension has three response categories, including no problems, some/moderate problems, or severe/extreme problems. The EQ-5D-5L, which will be used in this study, is a new version of the EQ-5D that includes five levels of severity in each of the existing five dimensions [34].

### Occurrence of adverse events

Adverse events (AEs) are undesirable and unintentional signs, symptoms, or diseases that appear during or after treatment in a clinical trial. Participants in this study will be required to voluntarily report any AEs. All AEs that occur during the trial will be documented. AEs that could occur in this study include skin irritation, bleeding, local hematoma, pallor, sweating or dizziness, fainting during acupuncture treatment, needle retention after treatment, continuous severe pain for more than 1 h after acupuncture treatment, or objective worsening of existing symptoms. The CRC will record all AEs in detail, including the time and date of occurrence, degree of severity, any measures related to treatment of the AE, and any potentially causal relationship between the trial and AE, as well as report all AEs to the PI and relevant IRB. In the case of SAEs, defined as those causing severe disability or malfunction, appropriate measures will be taken and the incident will be immediately reported to the PI and relevant IRB. If an AE is determined to occur because of the clinical trial, participants will notify the CRC and the PI and be compensated.

### Quality assurance

This protocol has been reviewed and revised several times by experts on acupuncture, neurology, statistics, and methodology. Before the trial, all researchers will be required to attend a series of training sessions, which will ensure that the personnel involved fully understand the trial protocol and standard operating procedures of the study. The Data Monitoring Committee (DMC) will comprise the PI, the CRC, and a researcher who are not involved in the data collection portion of the clinical trial. The DMC, which is independent from the sponsor and competing interests, will manage the data to ensure its validity. In the event that the protocol described herein is revised, the revisions will require approval from the IRB of DongShin University Gwangju Korean Medicine Hospital.

### Sample size estimation

We had no preliminary study or adequate previous studies upon which to base our estimate of sample size. Considering funding, the single-center study design, and time constraints, the maximum possible number of participants was set at 32; therefore, we adopted a pilot study design with eight participants in each of four groups.

As our study is a pilot study, the sample size will not be sufficient for determining the efficacy of acupuncture on MCI. Our study will indicate whether it is feasible to randomize participants to a trial of acupuncture treatment for MCI and suggest whether acupuncture is an acceptable treatment for the included participants.

### Statistical analysis

A statistician, who is not involved in data collection, will analyze the final data. We will perform a full analysis (FA) set to assess efficacy and a supplementary per-protocol (PP) analysis. We will compare the results of statistical analyses between the PP group and the FA group, and confirm whether there is a statistically significant difference between groups. If there is a significant difference between the PP and FA groups, the cause will be reviewed and reflected in the efficacy assessment. All statistical analyses will be performed using SPSS version 21 software.

Baseline characteristics will be described and compared among the groups. Continuous data will be presented as the mean and standard deviation, and compared using the independent *t* test or Wilcoxon rank-sum test. Categorical data will be presented as the frequency and percentage, and compared using the chi-square or Fisher’s exact test.

Within each group, changes in ADAS-K-cog, MoCA-K, CES-D, K-ADL, K-IADL, and EQ-5D-5L scores at 8 weeks (i.e., the end of the intervention) and 20 weeks (i.e., 12 weeks after the end of the intervention) after the start of the intervention will be analyzed relative to baseline scores using paired *t* tests or Wilcoxon signed-rank tests. The degree of change at each time point will be evaluated by repeated-measures analysis of variance and two-sample *t* tests or Wilcoxon rank-sum tests. Subanalyses will be performed according to participant age. All reported *P* values will be two-tailed with 95% confidence intervals. *P* < 0.05 will be considered statistically significant.

Missing values will be obtained using the last observation carried forward method. Interim analyses will not be performed.

### Confidentiality and data management

All identification records of the participants will be kept confidential. When the results of the study are published, the identification records will be accessed with IRB approval and the participants’ names will appear as initials to protect individuals’ identities, if necessary. All documents related to the trial, including case report forms (CRFs), will be recorded and labeled with participant identification codes and will not reveal the names of participants. The serial number codes will be stored in sealed, opaque envelopes and kept in a double-locked cabinet. All participant data will be recorded in Excel files by the CRC. All data will be checked by the CRC and rechecked by a researcher, both of whom are not included in this study’s data collection phase to ensure the reliability of the data. Continuous data will be coded as data values and categorical data will be coded as number sets for each item by a statistician who is not involved in the data collection phase of this clinical trial. The entry and coding of data will be double-checked. Electronic data will be stored on a password-protected computer. Anyone who is not approved by the IRB will not be able to access the data. In addition, raw data (i.e., CRFs) will be stored in a cabinet until the end of the study. Written informed consent for the publication of individuals’ details and accompanying images will be obtained from the participants.

## Discussion

Apart from needle insertion, factors such as needle sensation, psychological factors, acupoint specificity, needle duration, and acupuncture manipulation also influence the therapeutic effects of acupuncture [14]. Among these factors, acupoint specificity, needle duration, and acupuncture manipulation are objective. It is, therefore, necessary to identify the optimal acupoint specificity, needle duration, and acupuncture manipulation techniques in order to develop an optimal acupuncture treatment method for MCI. Although acupuncture has long been used to treat cognitive dysfunction, the optimal acupuncture treatment method for cognitive impairment has been understudied and is yet to be established. This study is expected to provide preliminary evidence for how to develop the optimal acupuncture treatment method for MCI.

For the acupoints and needle duration of the BA group, we adopted the acupuncture treatment procedures most commonly used in previous clinical trials on cognitive impairment and dementia [11, 12, 15]. According to the theory of traditional Chinese medicine (TCM), acupoint specificity is an important basis for clarifying the functionality of acupoints. The choice and compatibility of acupoints are considered to have a direct impact on the therapeutic effect [14]. The AS group in this study will receive acupuncture at KI3 in addition to the same treatment administered to the BA group. KI3 is one of the more frequently used acupoints and is reported to have a varying efficacy in the treatment of dementia [35]. Previous functional magnetic resonance imaging studies have indicated that acupuncture at KI3 can activate certain cognition-related regions [18–21].

Needle duration refers to the duration for which the needle is held at an acupoint after needle manipulation, with the aim of awaiting Qi arrival, regulating Qi circulation, eliminating pathogenic factors, and reinforcing antipathogenic Qi as well as the effect of manipulation. Different diseases require different durations of needle retention for treatment [14]. In the ND group in this study, we will adopt a 20-min needle duration, as described in a previous acupuncture study on cognitive impairment [22]. There are some methods of reinforcing or reducing acupuncture manipulations in TCM, which involve slow and quick needle manipulation and needle manipulation in concordance with the patient’s respiration. All manipulations are based on the principle of puncturing along and against the directional flow of the meridian Qi. Since stimulation gained by needle manipulation cannot be quantified, the EA group in this study will receive electrical stimulation, which was used in a previous acupuncture study on MCI [9].

In our study, cognitive function will be assessed using the K-MMSE and MoCA-K scales in the initial screening session. The MMSE is one of the more commonly used evaluation tools for cognitive function and the MoCA scale is a simple and highly sensitive cognition screening tool. Using the MMSE and MoCA scales for identifying possible cases of MCI is a first-step screening strategy for MCI [36]. We will use the ADAS-K-cog, MoCA-K, CES-D, K-ADL, K-IADL, and EQ-5D-5L scales for investigating the effects of different acupuncture treatment methods on cognitive function, depression, ADL, and quality of life in patients with MCI.

This protocol has some limitations. First, because of the lack of adequate preliminary studies and limited funding, this survey has been designed as a single-center pilot study with a small sample size. Second, while previous acupuncture studies on cognitive impairment have used body and scalp acupoints [22, 25, 26, 37], we will only use scalp acupoints for basic acupuncture treatment. Third, although all outcomes will be measured and recorded by an independent researcher in order to minimize the risk of detection bias, we cannot adopt a double-blind procedure because of the nature of acupuncture treatment.

### Dissemination policy

The authors will report the final data to the Ministry of Education, Republic of Korea, through the National Research Foundation of Korea. The authors will also publish the results after study completion.

## Trial status

This trial (protocol version number: DSACU, version 1.1; approved on September 17, 2018) is ongoing. Participant recruitment began on February 14, 2019 and is expected to be completed by the end of December 2020. The trial procedures are expected to be completed by the end of March 2021.

## Additional file

Additional file 1: SPIRIT 2013 Checklist: Recommended items to address in a clinical trial protocol and related documents (DOCX 368 kb)

## Data Availability

Not applicable; no data have yet been generated.

## Abbreviations

ADAS-K-cog: Korean version of the Alzheimer’s Disease Assessment Scale—cognitive subscale
ADL: Activities of daily living
AE: Adverse event
AS: Acupoint specificity
BA: Basic acupuncture
CES-D: Center for Epidemiological Studies Depression
CONSORT: Consolidated Standards Of Reporting Trials
CRC: Clinical research coordinator
CRF: Case report form
DMC: Data Monitoring Committee
EA: Electroacupuncture
EQ-5D-5L: European Quality of Life Five Dimension Five Level
FA: Full analysis
IRB: Institutional review board
K-ADL: Korean Activities of Daily Living
K-IADL: Korean Instrumental Activities of Daily Living
K-MMSE: Korean version of the Mini-Mental State Examination
MCI: Mild cognitive impairment
MMSE: Mini-Mental State Examination
MoCA-K: Korean version of the Montreal Cognitive Assessment
ND: Needle duration
NRF: National Research Foundation of Korea
PI: Principal investigator
PP: Per protocol
SAE: Serious adverse event
SPIRIT: Standard Protocol Items: Recommendations for Interventional Trials
TCM: Traditional Chinese medicine
